# 2D Electronics Based on Graphene Field Effect Transistors: Tutorial for Modelling and Simulation

**DOI:** 10.3390/mi12080979

**Published:** 2021-08-18

**Authors:** Bassem Jmai, Vitor Silva, Paulo M. Mendes

**Affiliations:** CMEMS-UMinho, University of Minho, 4800-058 Guimarães, Portugal; bassem.jmai@fst.utm.tn (B.J.); vitor.silva@inl.int (V.S.)

**Keywords:** graphene field-effect transistors (GFETs), MATLAB, Verilog, VHDL-AMS, modeling

## Abstract

This paper provides modeling and simulation insights into field-effect transistors based on graphene (GFET), focusing on the devices’ architecture with regards to the position of the gate (top-gated graphene transistors, back-gated graphene transistors, and top-/back-gated graphene transistors), substrate (silicon, silicon carbide, and quartz/glass), and the graphene growth (CVD, CVD on SiC, and mechanical exfoliation). These aspects are explored and discussed in order to facilitate the selection of the appropriate topology for system-level design, based on the most common topologies. Since most of the GFET models reported in the literature are complex and hard to understand, a model of a GFET was implemented and made available in MATLAB, Verilog in Cadence, and VHDL-AMS in Simplorer—useful tools for circuit designers with different backgrounds. A tutorial is presented, enabling the researchers to easily implement the model to predict the performance of their devices. In short, this paper aims to provide the initial knowledge and tools for researchers willing to use GFETs in their designs at the system level, who are looking to implement an initial setup that allows the inclusion of the performance of GFETs.

## 1. Introduction

The development of CMOS (complementary metal-oxide semiconductor) transistors is achieving its performance limit, due to the maximum downscaling according to Moore’s law. Several new materials have appeared with the potential to overcome silicon devices’ performance; most of these rely on two-dimensional (2D) materials. Those families of materials feature dangling-bond free surfaces exhibiting excellent electronic and optical properties. Such materials range from graphene to other 2D materials, such as the transition metal dichalcogenides (TMDCs). TMDCs have a finite bandgap, which is essential to enable low-power digital electronics. The state of the art of the TMDC transistors and silicon transistors is similar, as reported in [1], where MoS_2_ was used as the channel of the transistor, achieving an intrinsic maximum oscillation frequency (*f_max_*) of 50 GHz at low temperatures. Despite the promising results at cryogenic temperatures, the FET mobility at room temperature remains low for TMDCs. Because of that, graphene (a monolayer of carbon atoms in a honeycomb lattice) is being widely pursued as an enabling material; it has amazing properties at room temperature, such as high saturation velocity and high carrier mobility, which make this material suitable for radiofrequency (RF) applications, for example.

Since the discovery of graphene by Geim and Novoselov in 2004 [2], it continues to attract the interest of the scientific community, both for the prospects it offers in fundamental research [3,4], and for applied physics [5,6]. Graphene is a 2D material, consisting of a sheet of carbon atoms arranged in a honeycomb lattice, weakly bonded to a supporting substrate [7]. The absence of a band gap in the band structure of graphene [8] is a serious obstacle for the development of digital applications based on this material (in contrast with the TMDCs); nevertheless, this is not a limiting factor for analog applications. Given the broad potential of graphene, several GFETs (graphene field-effect transistors) have been reported in the literature; these range from biosensing applications to flexible electronics or radiofrequency circuits [9,10,11].

From initial measurements, performed on monolayer graphene, a very high mobility of about 20,000 cm^2^ V^−1^ s^−1^ was observed at low temperature (with high mobility also observed at room temperature), suggesting the possibility of the use of this material in very fast analog electronics, due to the very short carrier transit time. However, it must be noted that the properties of graphene—mainly its high mobility—change with the substrate where the graphene lays, as suggested in [12]. High mobility was achieved on suspended graphene on a SiO_2_ substrate, which suggests that high mobility can be achieved even after the graphene’s transfer to a substrate [13]. Despite this, suspended membranes of graphene are not suitable for the fabrication of GFETs, due to their non-compatibility with the conventional CMOS processes.

The good potential of graphene in GFETs—mainly in radiofrequency, where graphene’s high mobility is essential to achieve a good performance—is demonstrated by the cutoff frequency ($f_{T}$) of 100 GHz achieved in devices with a gate length of 240 nm in SiC substrates [14], or by the $f_{T}$ of 155 GHz in 40 nm gate length devices by PE-CVD-grown graphene transferred to DLC (diamond-like carbon substrates) [15]. Despite these promising results, research is still required, from different research fields, to achieve better performance in GFETs, as well to incorporate this technology at the system level. Thus, simple models that can help the researchers to predict the GFETs’ behavior need to be made available for integration in circuit-level tools.

In this paper, a review will be performed regarding the most common graphene field-effect transistors (GFETs) and their applications, along with discussion of how they can be modeled for use in tools that allow circuit- and/or system-level simulation, in order to assess the performance of electronic or sensing circuitry based on such GFETs. This paper is divided into four sections: firstly, a discussion of the different architectures for the graphene transistors is presented; then, the different fabrication techniques are presented and discussed; in the third section, the different substrates for GFET fabrication are addressed; finally, a model of the graphene transistor is presented, and its potential use discussed. This model was implemented in MATLAB, Verilog in Cadence, and VHDL-AMS in Simplorer, which are three tools widely used for electronic circuit simulation.

## 2. Most Common Graphene Transistor Topologies

A field-effect transistor is a device characterized mainly by its three terminals (four terminals, considering the bulk terminal): the drain, the source, and the gate. The drain and the source are interconnected by the graphene channel. The gate is isolated from the channel by a gate dielectric. The channel is characterized by its length (L) and its width (W). For a given drain-source voltage (V_DS_) a current (I_DS_) flows through the channel, from drain to source. By applying a potential (V_GS_) on the third terminal (the gate), this current can be modulated by adjusting the charge of the channel through the induced electric field—the basic operation of the field-effect transistors. A GFET is a particular implementation of the above description, where the channel is implemented using a graphene layer. 

**Figure 1:**
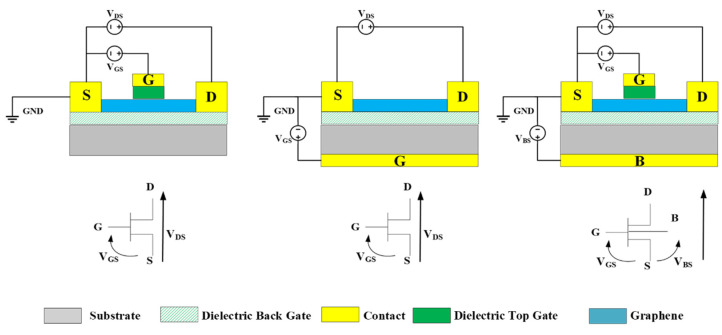
Most common topologies of graphene transistors. (**left**): Top-gated graphene transistor; (**center**): back-gated graphene transistor; (**right**): top-/back-gated graphene transistor.

This section is dedicated to describing the three most common topologies of graphene transistors. From the literature, as shown in Figure 1, we may identify three main types of GFET, classified essentially by the gate position: top-gated graphene transistor, back-gated graphene transistor, and top-/back-gated graphene transistor. 

The selection of one of the previous topologies will have implications for the fabrication methodology or for available applications, as well as for expected performance and modeling considerations. The next sections will address such issues, giving some insights about topology selection.

### 2.1. Top-Gated Graphene Transistor

Top-gated GFETs are widely found in publications related to RF applications [16,17,18], and are also very promising in the development of graphene-based liquid-gate transistors (SGFETs), with a wide range of applications in biosensors [19]. 

**Figure 2:**
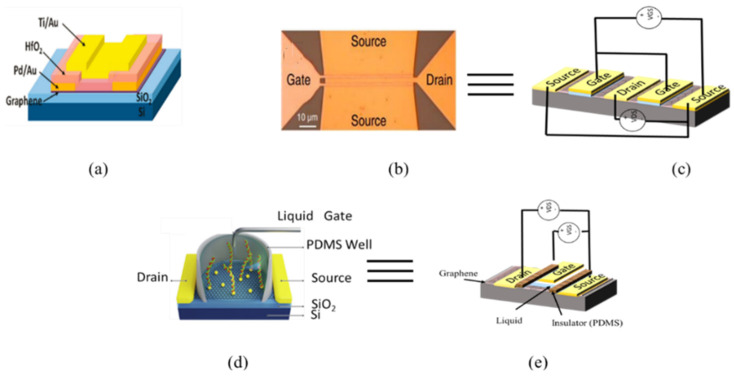
Top-gated graphene transistors: (**a**) single-gate [16]; (**b**) two-fingers [18]; (**c**) topology of the two-fingers transistor; (**d**) top-liquid gate [19]; and (**e**) topology of the top-liquid gate.

In Figure 2, three different examples of top-gated devices reported in the literature are shown. In Figure 2a, a simple case of a top-gated graphene transistor is presented. Figure 2b presents a top-two-fingers-gated device. In Figure 2c a double-top-gated equivalent topology is presented. In Figure 2d,e, a biosensor with a top-liquid gate and its architecture are presented, respectively. 

Top-gated graphene transistors are a suitable approach to applications where a thin oxide layer gate is required. This is necessary to exert more control over the electrostatically doped carriers in the channel and, consequently, a lower gate bias is required to modulate the channel. Despite all the advantages of this approach, the difficulty of growing an oxide on top of the graphene, without damaging its lattice and degrading its mobility, remains a problem. To solve this, several approaches have been developed, such as the physical transfer of a nanowire with an oxide shell to act as a gate electrode [16]. Standard fabrication processes are being researched to prevent the graphene lattice, such as the use of ALD (atomic layer deposition) to grow Al_2_O_3_, or the thermal growth of Al_2_O_3_ after the evaporation of a native layer of aluminum. Another approach could be the use of the boron nitride (h-BN) films to act as a gate dielectric. The h-BN film has a dielectric constant of ~3.4; however, due to the similarity with the hexagonal graphene’s structure, it is the suitable oxide to be used together with graphene due to the absence of dangling bonds on its surface, which makes this a chemically inert material. With this approach, the graphene’s mobility could be improved by one order of magnitude when compared with other approaches. In Table 1 several top-gated graphene devices are summarized. The reported devices consist of several devices that are under research to increase the transconductance ($g_{m}$), RF performance, or drain-current saturation of GFETs. As it is possible to observe in Table 1, the performance of top-gated graphene transistors may be increased by the correct choice of the gate dielectric, the self-alignment of the source and drain electrodes (which reduce the ungated region of the channel, and are very important to increase the RF performance), and by using complex gate structures (for example, the T-shaped gates, reducing the gate resistance). For biosensors, this approach enables the detection of lower concentrations of analytes. 

### 2.2. Back-Gated Graphene Transistor

**Figure 3:**
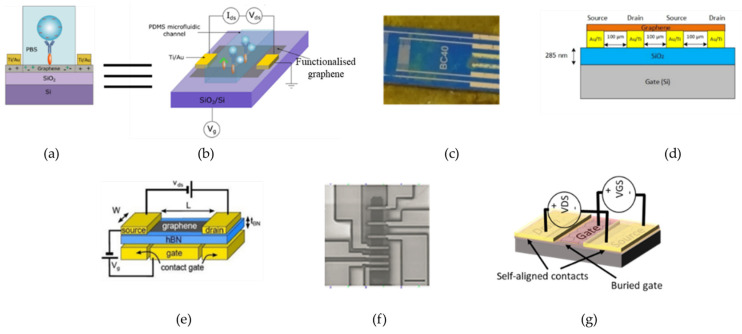
Back-gated graphene transistors: (**a**) graphene FET biosensor for the label-free sensing of exosomes [25]; (**b**) topology of the biosensor; (**c**) graphene photodetector [28]; and (**d**) the topology of the photodetector; (**e**) graphene FET used for the study of the saturation velocity with h-BN as a gate dielectric [29]; (**f**) graphene photodetector [30]; and (**g**) the topology of (**f**).

Another very widely used structure for GFET devices is the back-gated topology. From the literature, this group of GFETs is mostly used in biosensors [24,25] or photodetectors [26,27,28]. In this topology approach, the final device has the graphene of the channel exposed, which is very useful for sensors or for light collection. In this kind of device, the interaction of the graphene’s surface with light (photodetectors) or with molecules (biosensors) produces changes in graphene’s properties, which lead to a change in the graphene’s transfer curve, enabling the quantitative evaluation of the element that induced this change. However, with this topology, high transconductance is hard to achieve, given the small geometric capacitance of the gate. Typically, this kind of transistor is operated with high back-gate voltages, reaching dozens of volts, which is out of the range of the common applications of the electronic circuits. In Figure 3, some examples of back-gated devices are presented, being used as biosensors or photodetectors.

These devices (buried gates, in contrast with the back-gated devices) are very useful, mainly in RF applications, given the small achievable gate capacitance, reducing the parasitic capacitance, which increases the RF performance. In this approach, the common issues associated with the growth of an oxide on top of the graphene are avoided. In Table 2, several back-gated devices are reported and analyzed in terms of purpose and performance; they are mostly biosensors and photodetectors. Other devices are also present, where the focus of the device is the study of some of the transistor’s properties, and where the conservation of the graphene’s lattice is required. Devices where the h-BN films were used as back-gate dielectrics are also presented given their amazing properties when used together with graphene, as discussed earlier in this paper.

### 2.3. Top-/Back-Gated Transistor

**Figure 4:**
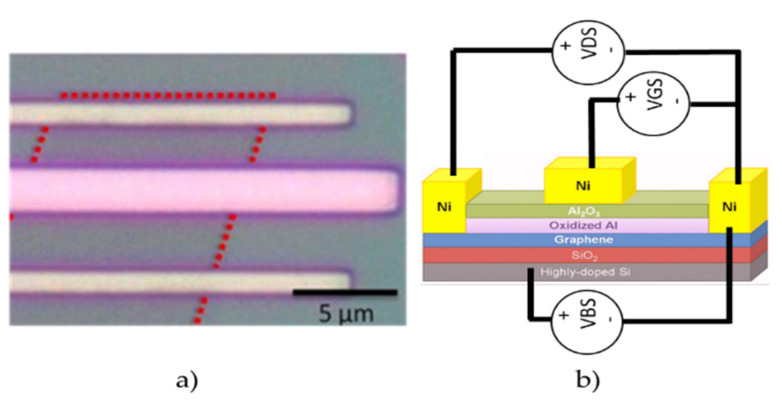
Top/back gated transistors: (**a**) GFET fabricated in [38]; and (**b**) its architecture.

For several applications, it is necessary to shift the Dirac point in order to electrostatically dope the graphene, adjusting the operation of the transistor. With this purpose, back gates are added to top-gated transistors. Figure 4 shows one top-/back-gated transistor and its topology. 

In fact, since graphene transistor research is only in the early stages, most of reported top-gated graphene transistors have back gates. Such devices are useful to tune all of the transistors to the same point (by tuning them all to the same Dirac point), reducing the variability between GFETs, which is a big drawback of GFETs today. In Table 3, several transistors with this configuration are presented. 

## 3. Fabrication Methods

After presenting a set of GFETs in each topology, briefly discussing their applications and performance, this section will present the issues related to the fabrication aspects that are necessary to consider while selecting one of the previous options to obtain 2D electronic circuits.

Despite the promising properties of GFETs, several issues need to be solved to increase the performance of such devices to the required levels, e.g., the low on/off ratio of the graphene devices is a big limitation on the use of these devices for logic applications [44]. However, despite the high carrier mobility of graphene, such mobility is highly affected by the surrounding materials and the growth method, such as the used polymer in graphene transfer or the gate oxide for GFET fabrication [45]. The high contact resistance between graphene and metals is also a limitation on the performance of GFETs, mainly for RF applications [34]. Finally, the access resistance (caused by the ungated zone between drain/source and channel) is also an important aspect in the fabrication of GFETs [46]. To ensure good performance in GFETs, a good graphene quality is necessary, as well as a fabrication methodology that preserves the graphene’s quality, while obtaining the device or the full system.

### 3.1. Graphene Fabrication Options

Table 4 shows different ways to obtain different GFET devices, with different topologies, where the graphene—the core material—was obtained via different techniques. Such techniques will have an impact on final device performance, fabrication complexity, and fabrication repeatability, as well as on cost.

Being the fundamental material to support the device characteristics, the methodology to obtain the graphene and transfer it to the fabrication steps has been widely explored. Several methods to obtain graphene have been developed, with the most common growing solutions being CVD on a metal foil [55], CVD on SiC substrates [56], and mechanical exfoliation from bulk material (graphite) [57].

**Figure 5:**
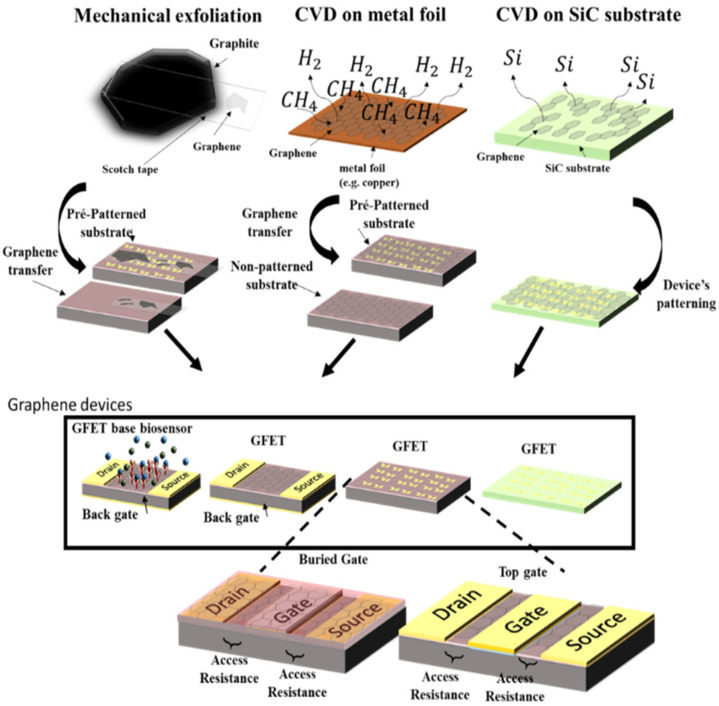
General fabrication steps of GFET-based devices.

In addition to the final performance considerations, the chosen method to obtain graphene constrains the GFET fabrication process and, eventually, the transistor’s topology. For example, when the graphene is obtained via mechanical exfoliation, the flake needs to be identified, and the structures of the source, drain, and gate are then fabricated [21], which make buried gated structures hard to fabricate, also limiting the scalability of the process. On the other hand, when CVD graphene is grown on metallic foils, since it needs to be transferred to the final substrate, and a large area could be covered by this kind of graphene, the structures of the source/drain and gate could be fabricated before or after the graphene transfer. However, degradation in the mobility of the graphene may be observed due to the transfer process [20]. In graphene grown on SiC substrates, the fabrication process is almost limited to top-gated structures [44]. Figure 5 summarizes the three main methodologies used to obtain the graphene for GFET fabrication.

The selection of the appropriate methodology will be dependent on the available processing methods at each facility, on the required application, and on the integration level and scalability desired.

### 3.2. Substrate for Graphene Transistors

Another relevant aspect that has an impact on the performance and/or fabrication steps, as well as integration potential, is the base substrate that is used to fabricate the GFET devices. The substrate may be imposed by the fabrication facilities, by the integration methodology required, or by the graphene fabrication and processing steps, with the latter being the topic of the next discussion.

Graphene transistors can be fabricated on several substrates, with the most common being Si/SiO_2_ [58], SiC [59], and glass and quartz [60]. Graphene transistors are also reported in flexible substrates such as polyethylene naphthalate (PEN) [60] or polyethylene terephthalate (PET) [61]. These are very useful in nonplanar applications (wearables for example), with their low insertion loss being a good characteristic for THz applications [62]. The most commonly used substrate is Si/SiO_2_, due to their compatibility with the standard fabrication processes and the easy optical identification of the graphene on SiO_2_. This property makes these the most suitable substrates for the fabrication of high-quality CVD-grown graphene devices after graphene transfer. The presence of a thick layer of SiO_2_ on top of the Si substrate makes this substrate also very useful in RF applications, decreasing the parasitic substrate capacitances. SiC substrates are often used for the epitaxial growth of graphene for electronics applications, with the advantages of the use of the conventional top-down lithography techniques well established in nanotechnology [60]. In contrast to CVD-grown graphene, graphene grown on SiC substrates does not require a transfer. However, SiC-grown graphene remains cost-ineffective. Insulator substrates such as glass or quartz are very useful in graphene FET fabrication. These substrates bring advantages for RF applications due to their ability to reduce the parasitic substrate capacitance, reducing the RF losses associated with the substrates [23].

## 4. Overview of Graphene Transistors

From the above analysis, it is possible to conclude that the fabrication of graphene transistors remains a challenging task. For different purposes, several aspects should be taken into consideration in order to ensure that the best design can be selected. Final performance can be a quantitative means of selecting a GFET solution, but there are other aspects to take into consideration. Table 5 presents, qualitatively, a few parameters that should be taken into consideration while evaluating which GFET to select. Since this type of analysis is always subjective, these are aspects to take into consideration while starting a new design. The definition of the evaluation criteria was based on [63], where a review of the nanofabrication processes was addressed, focusing on the issues experienced when working with graphene. Despite this being applied to biosensors, the issues are the same regarding the other applications (in optics, for example). The increased complexity of RF applications could be supported by works such as those reported in [17] or [32].

## 5. Performance Assessment Based on GFET Simulation

Before fabricating new devices or systems, it is highly desirable to have models that can predict the behavior of the devices, allowing, for example, the performance assessment of full electronic systems. Hence, this section will describe how to implement a model for monolayer/bilayer graphene transistors, using different tools, as well as how such a model can be used in DC analysis. Since the reported model describes the intrinsic behavior of the GFET, making the presented analysis valid for DC or low-frequency, adding external parasitic capacitances can further extend the model to specific applications, such as the performance of the GFET in high-frequency applications. The implemented model can be integrated in circuits such as frequency multipliers—a very common application of GFETs, used in many cases to validate the performance of the fabricated GFETs. More complex circuits can be simulated, such as inverters—a very important building block in logic circuits. The implemented model was obtained from [64,65]. In [65], a more detailed explanation is presented regarding the applications of the model. Despite the implemented model being optimized for top-/back-gated GFETs (the more complex GFET topology), it could be easily adapted to the other topologies by removing the parameters of the equation according to the structure (for example, for top-gated devices, removing all of the parameters that refer to the back-gate). This model was implemented in MATLAB, VHDL-AMS in Simplorer, and in Verilog to be used in Virtuoso from Cadence, for example. It should be noted that these models were made available online. 

### 5.1. GFET Transfer Characteristic Curve

**Figure 6:**
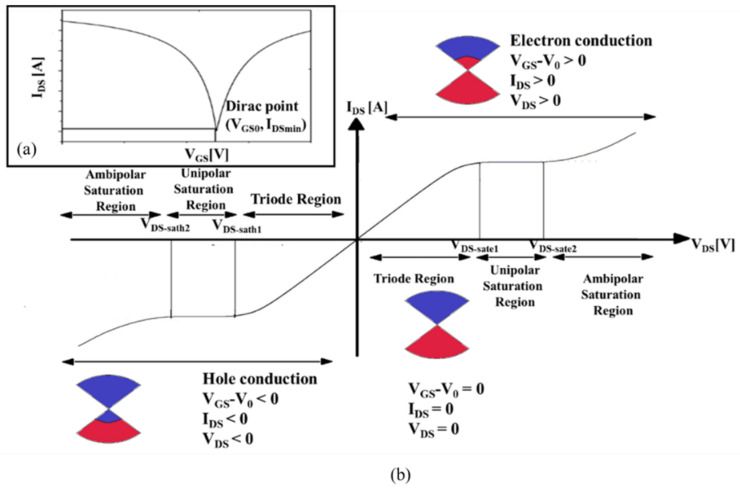
Graphene transistor characteristic curves: (**a**) I_DS_-V_GS_ characteristics when V_DS_ is constant; (**b**) I_DS_-V_DS_ characteristics.

The general I_DS_–V_GS_ characteristic curve, when V_DS_ is constant, is shown in Figure 6a. As shown in this figure, the Dirac point is the V_GS_ value were the GFET has its minimum I_DS_. As will be further shown, this point is obtained by setting V_GS0_ and V_BS0_ in Equation (2) (for top-/back-gated graphene transistors). These values are obtained empirically, extracted from the GFET transfer curve of the real device that is intended to be modulated. Figure 6b shows the general output characteristic curves (I_DS_–V_DS_) for a graphene transistor. It is possible to observe two regions in the figure: firstly, the conduction is performed through electrons, when V_GS_ > V_0_ with I_DS_ and V_DS_ positive, and secondly, conduction performed by holes, when V_GS_ < V_0_ with I_DS_ and V_DS_ negative. The electron conduction is composed of three regions—namely, the triode region, the unipolar saturation region, and the ambipolar saturation region—along with the charge interaction in the channel. For hole-based conduction, these three regions are also present. These DC characteristic curves can be obtained by using the implemented model, which indicates the correct behavior of the model and its ability to be useful, mainly in DC simulations, as will be shown in the next section. 

### 5.2. GFET Simulation Platforms

**Figure 7:**
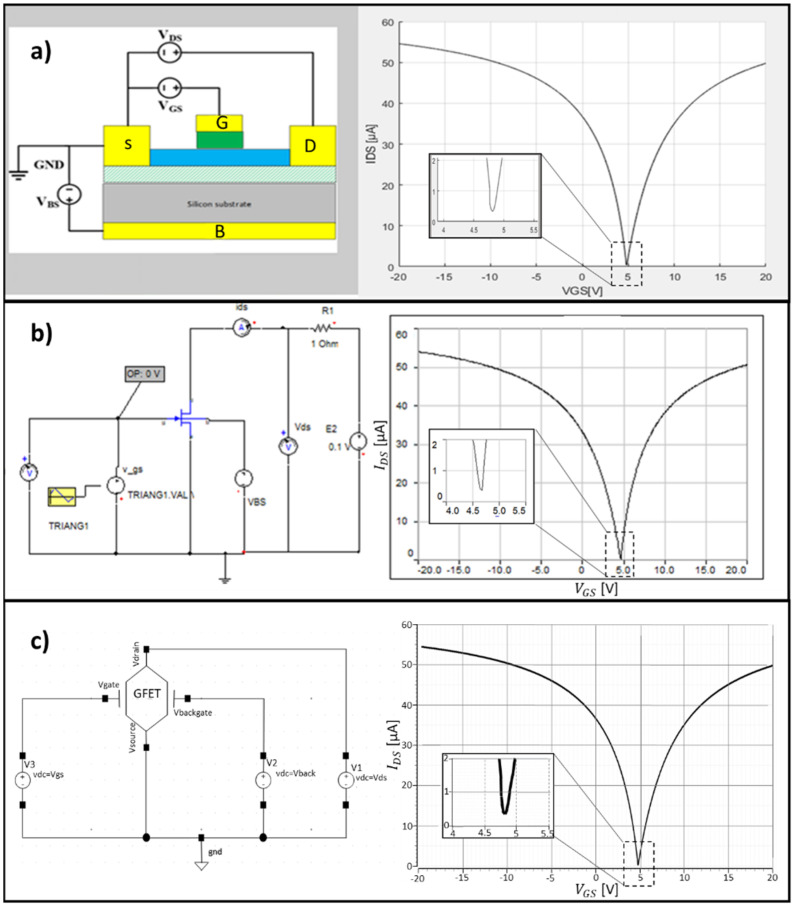
Interface of the GFET platforms: (**a**) MATLAB; (**b**) Simplorer; and (**c**) Cadence. The results presented for the GFET transfer characteristic were obtained from [64].

As was previously mentioned, this model was implemented in MATLAB, VHDL-AMS in Simplorer, and in Verilog to be used in Virtuoso from Cadence. The models are available as Supplementary Material S1. These languages were selected since they are a set of tools widely used by the multidisciplinary research community in this field. Since this paper intends to be used in different areas of research, from electronics to health sciences, the authors consider them to be the most appropriate. MATLAB was chosen because of its wide application in different areas such as nanotechnology or life sciences, allowing the researchers to easily integrate our platform with their models. The selection of VHDL-AMS and Verilog languages (hardware description languages, used to model electronic systems following the proprietary language standardized in IEEE: standard IEEE 1076.1999 for VHDL-AMS, and the IEEE 1364.2001 for Verilog) were selected to provide tools for circuit designers to integrate graphene transistors in their circuits, exploiting the full potential of graphene technology, potentially leading to the development of new devices. In this section, those platforms where the model was implemented will be presented. These models are available in the Supplementary Materials. A small tutorial of how to apply this model is presented. In Figure 7, it is possible to observe the tree interfaces developed under this work.

**Figure 8:**
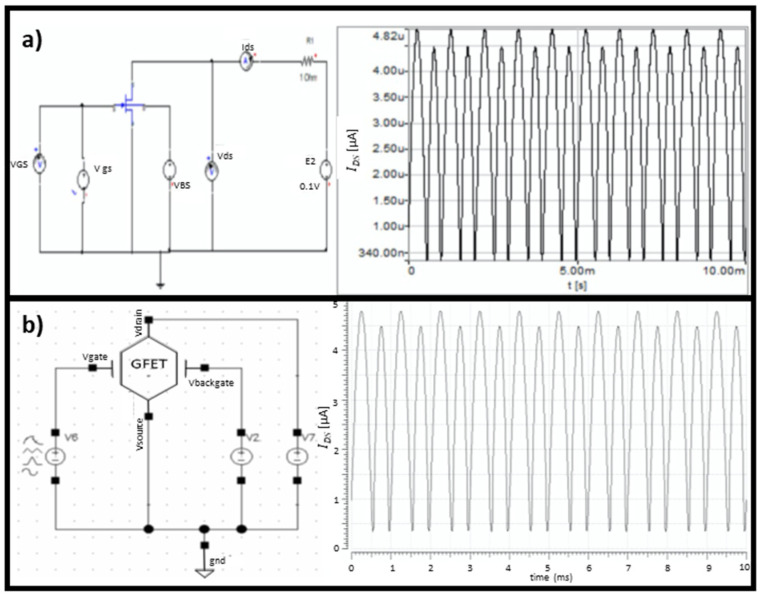
Frequency doubler simulated in (**a**) Simplorer and (**b**) Cadence. The graphs show the drain-to-source current output for a sine wave input (VGS) with a frequency of 1 kHz, an amplitude of 300 mV, and an offset of 4.9 V.

Among the several possible applications of GFETs, one is to use this device as a frequency doubler, due to the V-shaped I–V output characteristic curve of GFETs. To show the correct behavior of the model, a frequency doubler was implemented using Simplorer and Cadence, as shown in Figure 8, where a sinusoidal wave of 1 kHz was used as an input signal.

As can be shown, since the GFET rectifies the input signal, the output signal is a wave with new frequency components—namely, the double frequency. In this way, it is shown that we can use this model to rectify a signal, or as a multiplier.

Despite the graphical aspects related to the model display, which are straightforward in each tool, it is necessary to define a solution to implement the model for each desired GFET (how to set the specific GFET parameters into the model), as well as to define how the calculations will be performed.

**Figure 9:**
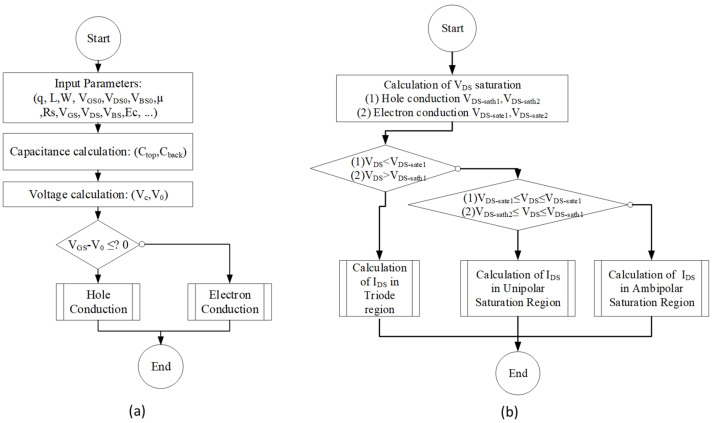
Flowchart of graphene transistor model: (**a**) Principal code (Level 0); (**b**) (1) hole and (2) electron conduction functions.

Figure 9 presents a flowchart of the implemented model of the graphene FET, showing the steps that must be implemented to compute the required voltages and currents. Figure 8a presents the principle of the modeling of the graphene transistors in DC mode. Firstly, the user sets the physical and geometric input parameters. According to this, the next parameters should be provided by the user, such as the electric charge (*q*), substrate thickness (*Hsub*), top-gate dielectric thickness (*tox*), channel width (*W*), gate length (*L*), back-gate dielectric constant at the Dirac point (*V_BS0_*), top-gate voltage at Dirac point (*V_GS0_*), charge density (*n_top_*), critical electric Field (*Ec*) and electron/hole mobility (µ), back-gate dielectric thickness (Hsub), top-gate dielectric constant (*ℇt*), series resistance (Rs), and the reduced Planck’s constant (h). As soon as the user writes all the necessary data, as a first step, the program must calculate the capacitances and the voltages. After that, a comparison between V_GS_ and V_0_ (threshold voltage) is performed to select the adequate conduction region, performed by holes or electrons. The surface potential of the channel (V_c_) is also calculated. Then, a validation is performed to evaluate in which region the GFET is being operated (as shown in Figure 9b). The variables addressed in the flowchart represented in Figure 9 will be explained in the next section.

### 5.3. GFET Model Parameters Computation

After the explanation of use, and about the parameters that can be modified in each model to compute the GFET characteristics, the equations that define the transistors will be presented.

To model the top-/back-gated transistor, it is necessary to compute the capacitances of the device—namely, the effective gate capacitance (*C_top_*), the back-gate capacitance (*C_back_*), and the channel capacitance, which are dependent on the quantum capacitance (*C_q_*) and the capacitance between gate and channel (*C_e_*). Those parameters are calculated following the expressions in Equation (1) [64]:

(1) $$\{\begin{array}{c} C_{top}=\frac{Ce.Cq}{Ce+Cq}\\ C_{back}=\varepsilon_{r}\frac{K_{sub}}{H_{sub}} \end{array}with\{\begin{array}{c} C_{q}=q^{2}\sqrt{\frac{n_{top}}{\pi }}\\ C_{e}=\varepsilon_{r}\frac{k}{t_{ox}} \end{array}$$

The surface potential of the channel (*V_c_*) depends on the critical electric field (*Ec*) and the gate length (*L*), while the threshold voltage (*Vo*) depends of the gate voltage at the Dirac point (*V_GS0_*), the capacitance between the back gate and the channel (*C_back_*), the effective gate capacitance (*C_top_*), the back-gate voltage at the Dirac point (*V_BS0_*) and the back-gate voltage (*V_BS_*). These relationships are presented in Equation (2):

(2) $$\{\begin{array}{c} V_{c}=E_{c}.L\\ V_{0}=V_{GS0}+\frac{C_{back}}{C_{top}}(V_{BS0}-V_{BS}) \end{array}$$

As previously mentioned, to select the region where the graphene FET is being operated, a comparison between the gate voltage (*V_GS_*) and the threshold voltage (*V_0_*) must be performed. If (*V_GS_* > *V_0_*), the transistor is working via hole conduction; otherwise, the transistor works in electron conduction.

Figure 8b shows the sub-function diagram of the hole conduction and the electron conduction. For both, this function starts with the calculation of *V_DS-sat1_* and *V_DS-sat2_* for the hole conduction and the electron conduction, as shown in Equation (3) [65]:

(3) $$\{\begin{array}{c} V_{ds-sat1}=\frac{1}{{\left(1+\alpha \right)}^{2}}\left(2V_{0}\alpha \left(1+\alpha \right)+(\alpha -1)\left(V_{c}-\sqrt{V_{c}^{2}+2V_{c}V_{0}\left(\alpha +1\right)}\right)\right)\\ V_{DS-sat2}=V_{DS-sat1}+\frac{1}{2}\left|V_{0}-V_{DS-sat1}\right| \end{array}$$

where $\alpha =\beta W\mu C_{top}R_{s}$, with 1 < *β < 1.4* as a constant to help in the fitting of real data.

The output current is presented in Equation (4) for the different regions [64,65]:

(4) $$I_{DS}=\{\begin{array}{c} \frac{1}{4R_{S}}\left(V_{c}-V_{DS}+2\alpha \left(\frac{V_{DS}}{2}-V_{0}\right)-\sqrt{{\left(V_{c}-V_{DS}+2\alpha \left(\frac{V_{DS}}{2}-V_{0}\right)\right)}^{2}-4V_{c}V_{DS}}\right)—\mathrm{Triode}\;\mathrm{Region}\\ \frac{\alpha }{R_{s}{\left(1+\alpha \right)}^{2}}\left(-V_{c}+\left(1+\alpha \right)V_{0}+\sqrt{V_{c}^{2}-2(1+\alpha )V_{c}V_{0}}\right)\;—\mathrm{Unipolar}\;\mathrm{Saturation}\;\mathrm{Region}\\ \begin{array}{l} \frac{\alpha }{R_{s}{\left(1+\alpha \right)}^{2}}\left(-V_{c}+\left(1+\alpha \right)V_{0}+\sqrt{V_{c}^{2}-2(1+\alpha )V_{c}V_{0}}\right)+\frac{W}{2L}\mu_{n}C_{top}V_{DS-sat2}^{2}\left(\frac{V_{DS}}{V_{DS-sat2}}-1\right)\;\\ —\mathrm{Ambipolar}\;\mathrm{Saturation}\;\mathrm{Region} \end{array} \end{array}$$

## 6. Conclusions

In this paper, an overview of different graphene transistor topologies was presented, looking for the most common gate topologies present in the literature, and taking into consideration the base substrate and the fabrication technology. It was found that top-gated transistors are more widely used for applications where the fine control of the graphene channel is required, such as high-frequency applications. Back-gated devices, including the buried gated devices, were found to be widely used for applications in sensors, photodetectors, and biosensors, due to the full exposure of the graphene to the entity that will be transduced. Buried gates are very useful in RF applications, presenting the advantages of top-gated devices while reducing the parasite capacitances, which are a known limiting factor in the RF performance of the devices. The top-/back-gated devices are very useful due to the addition of the possibility of the tuning of the Dirac point by adding a back gate to the top-gated devices, useful to set different transistors for approximately the same work regions at the same voltages, reducing the variability between devices. 

Graphene FETs are generally fabricated using Si/SiO_2_ as a substrate, due to its advantageous compatibility with standard fabrication processes. Another substrate that is used is SiC, which has the advantage that graphene developed on SiC substrates does not require the transfer steps, and can be used in high-temperature applications. The other types of insulating substrates, such as glass or quartz, have the advantage of allowing a much-reduced parasitic capacitance, which decreases the RF losses associated with the substrates. Regarding the graphene growth, despite the better quality of exfoliated graphene from graphite, CVD-grown graphite is the preferred method, since it has a good quality and enables the growth of large areas, being suitable for large-scale fabrication, while the epitaxial graphene grown on SiC remains expensive. Thus, it is possible to conclude that the fabrication of graphene transistors remains challenging. For different purposes, several aspects should be taken into consideration in order that the best design can be selected. In general, for RF applications, top-gated transistors or buried gates are preferable, since they enable better control of the graphene’s channel. For sensors (biosensors and photodetectors), the most common topology reported in the literature is back-gated devices, due to the existence of an exposed graphene gate owing to its easy functionalization, as well as allowing for liquid top-gated devices.

Finally, this paper presents a tutorial to be used in simulation tools and to allow the performance assessment of GFETs using a model of graphene transistors using MATLAB, VHDL-AMS, and Verilog. Those models are available in the Supplementary Materials to be used in MATLAB, Simplorer, and Virtuoso from Cadence.

## Figures and Tables

**Table 1:** Top-gated graphene transistors.

Purpose	Graphene Type	Differentiating Approach	Performance	Ref
Achieve high transconductance and drain-current saturation in GFETs	CVD	Self-aligned source/drain electrodes	Maximum $g_{m}$ of 250 µS/µm	[16]
GFET for RF applications	Mechanical exfoliation	Nanowire as the gate of the graphene transistor; self-aligned source/drain electrodes	Maximum $g_{m}$ of 1.27 mS/µm Intrinsic *f_T_* $f_{T}=$300 GHz	[17]
Improve the drain-current saturation increasing the gain in RF transistors	CVD	Thin $Al_{2}O_{3}$ gate oxide dielectric (~4 nm)	$\frac{f_{max}}{f_{T}}\;$> 3 $A_{v}>30\;\mathrm{dB}$	[18]
DNA biosensor	CVD	Use of a liquid gate and PDMS well to isolate source/drain electrodes from the liquid gate	Detection of full hybridization of the complementary strand down to 15 aM	[19]
Graphene RF power amplifier	Grown by CVD on SiC substrate	Dual top gate	Power amplifier gain of 8.9 dB at 2.5 GHz	[20]
GFET for RF applications	Mechanical exfoliation	Dual top gate	Maximum $g_{m}$ of 550 µS/µm Intrinsic$f_{T}=$14.7 GHz	[21]
Study of the low-temperature $SiN_{x}$ deposition of gate oxides in GFETs	Grown by CVD on SiC substrate	Low temperature deposition of $SiN_{x}\;$ by Cat-CVD	No gate current leakage	[22]
GFET for RF applications	CVD	T-shaped gate and drain/source; self-aligned source/drain electrodes	Intrinsic $f_{max}=200\;\mathrm{GHz}$ Extrinsic $f_{max}=106\;\mathrm{GHz}$	[23]

**Table 2:** Back-gated graphene transistors.

Purpose	Graphene Type	Differentiating Approach	Performance	Ref
High-sensitivity label-free DNA biosensors	CVD	Electrolysis bubbling method for graphene transfer Annealing in Ar/$H_{2}\;$ before functionalization	Detection limit depends on the length of the DNA, for 60-mer DNA the detection limit is 1 fM.	[24]
Graphene FET biosensor for the label-free sensing of exosomes	CVD	Back-gate contact made with silver conductive paint	Exosome detection of at least 0.1 μg/mL	[25]
Fabrication of graphene frequency multipliers	Mechanical exfoliation	Back gate on doped silicon wafer	Able to work with 10 kHz input frequencies	[26]
Photodetectors	CVD	Multilayer graphene to transport charges and to absorb light.	Photodetection from the visible to the mid-infrared range, with mid-infrared responsivity higher than 1 AW^−1^	[27]
Substrate effects on GFET photodetectors	Electrochemical delamination	Use of different semiconducting substrates	----	[28]
Study of velocity saturation graphene FETs: design and performance	Pulsed CVD	Use of the h-BN as a gate oxide. Dual-gate device for RF characterization.	$\frac{f_{max}}{f_{T}}\;$> 5 in Zenner–Klein regime	[29]
Achieve a high $f_{max}$	CVD	Buried gates with depth-to-with ratio up to sixfold to reduce gate resistance	Intrinsic $f_{T}=$35 GHz and $f_{max}=50\;\mathrm{GHz}$	[30]
Increase the transconductance for high-speed biosensors	CVD	Exposed graphene to enable the functionalization with biomolecules	Extrinsic $f_{T}=$22 GHz and $f_{max}=11\;\mathrm{GHz}$ $g_{m}$= 16 mS	[31]
RF graphene transistor with a high $f_{max}$	CVD	T-gate structure to reduce the gate resistance	Extrinsic $f_{T}=$11.4 GHz and $f_{max}=15\;\mathrm{GHz}$	[32]
Comparison between top and buried gates and their effect on fringing capacitance	CVD	Self-aligned buried gates	Low fringing capacitance in buried gates	[33]
Graphene integrated frequency multiplier	CVD	Inverted T-gate to reduce the gate resistance and development of an IC using a 200 nm platform	Frequency multiplayer gain of ~−25 dB at an input of 1 GHz	[34]
GFET with h-BN as a gate dielectric and support material for GFETs	---	h-BN as a gate dielectric	Intrinsic transconductance above 400 mS/mm	[35]
Improvement of the process-induced mobility degradation of graphene	CVD	Development of buried bottom gates	$I_{on}/I_{off}$ ratio of 5.31 Maximum $g_{m}$ of 6.85 µS/µm Intrinsic $f_{T}=$2 GHz and $f_{max}=13\;\mathrm{GHz}$	[36]
Photodetectors	Grown by CVD on SiC substrate	Back gate to modulate graphene conductivity	Non-local, position-sensitive, and large-area photodetection.	[37]

**Table 3:** Top-/back-gated graphene transistors.

Purpose	Graphene Type	Fabrication Techniques	Performance	Ref
GFET	Mechanical exfoliation	Gate oxide (Al_2_O_3_) deposited by ALD viaprior metallization of Al.	Preservation of the graphene mobility after the gate dielectric deposition (8000 cm^2^/Vs)	[38]
GFET top-gated-based frequency doubler	Mechanical exfoliation	Yttrium oxide as a gate dielectric	Able to work with 200 kHz input frequencies	[39]
GFET fabricated with HCa_2_Nb_3_O_10_ nanoflakes as a gate dielectric	Mechanical exfoliation	Use of HCa_2_Nb_3_O_10_ nanoflakes	High top-gate capacitance, small top-gate current leakage	[40]
GFET with high I_on_/I_off_ ratio and large transport bandgap	Purchased bilayer graphene (mechanical exfoliation)	Gate oxide of HfO_2_ deposited by a prior deposition of an organic seed	I_on_/I_off_ ratio≈100 Electrical band gap > 130 meV	[41]
GFET to extract the carrier mobility of graphene	Mechanical exfoliation	Yttrium oxide as gate dielectric	--------	[42]
GFET for RF electronics	Mechanical exfoliation	h-BN used as top and back gate dielectric	Current density of 1.2 A/mm Extrinsic f_T_ = 33 GHz	[43]

**Table 4:** Fabrication techniques used for graphene transistors.

Purpose	Graphene Type	Ref
CVD using metal foil substrate	Top	[47]
	Back	[48]
	Back	[49]
CVD on SIC	Top	[50]
	Top	[51]
	Back	[52]
Mechanical exfoliation	Top	[38]
	Back	[53]
	Back	[54]

**Table 5:** Qualitatively evaluation of GFETs regarding topology, substrate or fabrication complexity (evaluation criteria from adequate/complex to inadequate/less complex, +++, ++, +−,−).

Application	Topologies			Substrate			Fabrication Complexity
	Top-Gate	Back-Gate	Top-/Back-Gate	Si/SiO_2_	SiC	Quartz/Glass	
RF applications	+++	+++	+++	++	++	+++	+++
Biosensors	−	+++	+−	+++	++	+−	+++
Optics	−	+++	−	++	++	+++	++

## Supplementary Materials

The following are available online at https://www.mdpi.com/article/10.3390/mi12080979/s1, Models S1: Matlab, Verilog, and VHDL-AMS models.
